# Parents exposed to warming produce offspring lower in weight and condition

**DOI:** 10.1002/ece3.9044

**Published:** 2022-07-17

**Authors:** Rachel K. Spinks, Jennifer M. Donelson, Lucrezia C. Bonzi, Timothy Ravasi, Philip L. Munday

**Affiliations:** ^1^ ARC Centre of Excellence for Coral Reef Studies James Cook University Townsville Queensland Australia; ^2^ Division of Biological and Environmental Sciences and Engineering, Red Sea Research Center King Abdullah University of Science and Technology Thuwal Saudi Arabia; ^3^ Marine Climate Change Unit Okinawa Institute of Science and Technology Graduate University Onna Japan

**Keywords:** climate change, coral reef fish, maternal effects, ontogenetic timing, paternal effects, transgenerational plasticity

## Abstract

The parental environment can alter offspring phenotypes via the transfer of non‐genetic information. Parental effects may be viewed as an extension of (within‐generation) phenotypic plasticity. Smaller size, poorer physical condition, and skewed sex ratios are common responses of organisms to global warming, yet whether parental effects alleviate, exacerbate, or have no impact on these responses has not been widely tested. Further, the relative non‐genetic influence of mothers and fathers and ontogenetic timing of parental exposure to warming on offspring phenotypes is poorly understood. Here, we tested how maternal, paternal, and biparental exposure of a coral reef fish (*Acanthochromis polyacanthus*) to elevated temperature (+1.5°C) at different ontogenetic stages (development vs reproduction) influences offspring length, weight, condition, and sex. Fish were reared across two generations in present‐day and projected ocean warming in a full factorial design. As expected, offspring of parents exposed to present‐day control temperature that were reared in warmer water were shorter than their siblings reared in control temperature; however, within‐generation plasticity allowed maintenance of weight, resulting in a higher body condition. Parental exposure to warming, irrespective of ontogenetic timing and sex, resulted in decreased weight and condition in all offspring rearing temperatures. By contrast, offspring sex ratios were not strongly influenced by their rearing temperature or that of their parents. Together, our results reveal that phenotypic plasticity may help coral reef fishes maintain performance in a warm ocean within a generation, but could exacerbate the negative effects of warming between generations, regardless of when mothers and fathers are exposed to warming. Alternatively, the multigenerational impact on offspring weight and condition may be a necessary cost to adapt metabolism to increasing temperatures. This research highlights the importance of examining phenotypic plasticity within and between generations across a range of traits to accurately predict how organisms will respond to climate change.

## Graphical abstract

Our study teases apart the timing of maternal and paternal non‐genetic contributions to offspring phenotype in response to warming in a coral reef fish. We found offspring were lower in body weight and physical condition when parents were exposed to elevated water temperature, regardless of when or which parent was exposed. Yet the parental thermal experience did not impact offspring sex ratios. We discuss the ecological consequences of these results in comparison to other ectotherms.

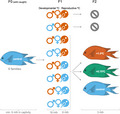

## 1 INTRODUCTION

Rapid environmental change poses a threat to biological systems through effects on the phenotypic traits of individual organisms that influence population sustainability. Smaller body size, reduced physical condition, and skewed sex ratios are common responses of ectotherms to global warming (Geffroy & Wedekind, 2020; Reading, 2007; Sheridan & Bickford, 2011). Reduced size and condition at higher temperatures are often due to increased metabolic rates alongside an inability to compensate with greater food intake or reallocate energy (Sheridan & Bickford, 2011). For marine fishes, a 20% reduction in assemblage‐averaged maximum body weight has been predicted by 2050 owing to warming, which has ramifications for ecosystem productivity and fisheries harvest potential (Cheung et al., 2013). Shrinking body size with decreasing latitude is a commonly observed pattern in nature, suggesting a reduced size may be adaptive owing to increased thermal tolerance (Angilletta Jr et al., 2004; Forster et al., 2012; Leiva et al., 2019; Verberk et al., 2020; but see Audzijonyte et al., 2020) However, reduced body size and condition can increase predation risk, reduce fecundity, and decrease competitive ability (Blueweiss et al., 1978; Booth & Hixon, 1999; Goatley & Bellwood, 2016; Grorud‐Colvert & Sponaugle, 2006; Meekan et al., 2006; Poulos & McCormick, 2015). Sex ratios are also an important component of population sustainability since reproduction typically depends on the availability of males and females. Increased temperatures can bias sex ratios in reptiles, and to a lesser extent amphibians and fishes, owing to temperature‐dependent sex determination during early development (Bickford et al., 2010). Population growth is often constrained by female fecundity (Hill et al., 2000; Morales et al., 2005), so in species where increased temperatures lead to a male bias, like fishes (Geffroy & Wedekind, 2020), warming can pose a threat to population replenishment. Yet, organisms may be able to maintain size, condition, or sex ratios in a future warm world through phenotypic plasticity (non‐genetic response to environmental variation; Pigliucci, 2001) (Donelson & Munday, 2015; Salinas & Munch, 2012; Shama, 2015). Plasticity is predicted to be especially important in responding to rapid climate change because it typically operates over a much faster timescale than adaptation by natural selection (Geoghegan & Spencer, 2012; Klironomos et al., 2013).

The environment may induce phenotypic change both within a single generation (within‐generation plasticity) and across generations (parental effects or transgenerational plasticity). Parental effects occur through the transfer of non‐genetic information via epigenetic mechanisms (e.g., DNA methylation, histone modification, or small non‐coding RNAs), cell structures, hormones, nutrients, or behaviors (Bonduriansky et al., 2012; Ho & Burggren, 2010). Parents may anticipate offspring conditions in order to produce progeny with the best phenotype for that environment (Donelson & Munday, 2015; Marshall & Uller, 2007; Shama & Wegner, 2014). Defined as anticipatory parental effects, they are considered adaptive when offspring performance improves in the environment that is predicted by the parental environment (Burgess & Marshall, 2014). Conversely, anticipatory parental effects may be maladaptive when the parental environment induces phenotypic change in the offspring but it does not match the local offspring environment, consequently decreasing offspring fitness. The risk of a mismatch between the anticipated and actual environment will tend to select against anticipatory parental effects and may explain the weak evidence across taxa (Bonduriansky & Crean, 2018; Radersma et al., 2018; Sánchez‐Tójar et al., 2020; Uller et al., 2013). By contrast, carry‐over parental effects—where the parental environment influences offspring phenotype regardless of the offspring environment—are likely widespread because they are not contingent on environmental predictability and, therefore, do not require complex machinery to assess environmental conditions and adjust offspring phenotypes accordingly (Bonduriansky & Crean, 2018; Jablonka et al., 1995). Carry‐over parental effects can be adaptive since the transfer of a high parental condition to offspring would be beneficial in many circumstances (Bonduriansky & Crean, 2018; Jablonka et al., 1995); however, they may also be maladaptive (Evans et al., 2017; Marshall & Uller, 2007; Valdivieso et al., 2020) when a low parental condition is passed on (but see positive net selection argument in Bonduriansky & Crean, 2018). Therefore, in order to predict the effect of future warming on ectotherms, it is necessary to understand whether plasticity within and between generations may mitigate or exacerbate the negative effects of warming.

Parental effects may derive from mothers, fathers, or both parents. Maternal effects are generally assumed to be more important than paternal effects owing to the mother's role in embryonic nutritional provisioning and the transfer of mitochondria (Ghiselli & Milani, 2020; Mousseau & Fox, 1998). However, this classic idea is a simplistic view of maternal and paternal contributions, with both parents often having a genetic (i.e., DNA) and nongenetic (e.g., epigenetic) influence on offspring phenotypes (Bonduriansky & Day, 2009). Furthermore, paternal provisioning (e.g., nuptial gifts or substances for embryos) and care may increase selection for paternal effects (Griffith et al., 1999; Hunt & Simmons, 2000; Smedley & Eisner, 1996). Maternal or paternal effects may evolve under sex‐specific reproductive strategies, socializing, foraging, predation, or parasitism (Burke et al., 2020; Hellmann et al., 2020; Lewis et al., 2002; Magnhagen, 1991; Ruckstuhl, 2007; Zuk & McKean, 1996). But even when the sexes are alike, it is possible that mothers and fathers experience different environments when temporal environmental variation exists and breeding pairs are of mixed age (Mills, 1973) or large spatial areas are traversed (Shimada et al., 2020), thereby leading to the potential for differing maternal and paternal effects.

Whether maternal and/or paternal effects occur may also depend on the ontogenetic timing of parental exposure, with early periods in development most sensitive to environmental change (Donelan et al., 2020; West‐Eberhard, 2003). For example, developmental exposure to stressful conditions, such as a heatwave, can allow individuals to cope better with those same conditions later in life and this benefit may be passed to offspring (Donelson, Munday, McCormick, et al., 2012). By contrast, parents that reproduce during stressful conditions may have insufficient resources for their offspring, resulting in negative parental effects (Donelson et al., 2016; Fuxjäger et al., 2019; Radersma et al., 2018). Currently, great interest exists for research on plasticity in a climate change context (Donelson et al., 2018; Gunderson & Stillman, 2015; Reusch, 2014; Seebacher et al., 2015); however, owing to the logistical challenges, few attempts have been made to disentangle the ontogenetic timing of maternal and paternal effects. Examining time‐ and sex‐specific parental effects will provide greater mechanistic insight of plasticity and enhance our capacity to predict whether plasticity may help tropical ectotherms cope with warming.

Here, we investigated the ontogenetic timing of paternal, maternal, and biparental exposure to elevated temperature on offspring size, condition, and sex ratios in a coral reef damselfish, *Acanthochromis polyacanthus* (Bleeker 1855). Specifically, males and females from six families developed from hatching in a present‐day average temperature for their population (control), or 1.5°C above the average temperature, consistent with climate change projections and marine heatwaves that already occur (Frölicher et al., 2018; IPCC, 2019). Once mature (1.5 years), the fish were divided orthogonally into control and elevated reproductive temperatures and breeding pairs were created such that every thermal combination of sex and time (development, reproduction, or both) occurred (eight parental treatments). Offspring from these breeding pairs were reared at the present‐day average summer temperature (control), +0.75°C and + 1.5°C for three months, at which time offspring standard length, weight, condition, and sex ratios were measured. Fulton's K condition factor was used to estimate body condition due to its wide use in fishes (Froese, 2006), particularly coral reef fishes (e.g., Green & McCormick, 2005a, 2005b; Grorud‐Colvert & Sponaugle, 2006; Kingsbury et al., 2020). This factor assumes heavier fish of a given length are in better physical condition and it may correlate with muscle and liver energy content and fecundity (Lambert & Dutil, 1997, 2000; Neff & Cargnelli, 2004). Our experimental design allows estimation of the relative non‐genetic maternal and paternal contributions, parental timing effects, within‐generation plasticity, and family‐level (i.e., mostly genetic) effects. The life history, reproductive strategy, and high site fidelity of *A*. *polyacanthus* suggest temporal variation would most likely explain differing parental thermal histories in natural populations, such that in mixed‐age pairs one parent may have developed during a marine heatwave, the other during a year of usual sea temperature, and they then bred during a heatwave. We hypothesized that anticipatory parental effects were likely to occur because the parental environment could be predictive of the offspring environment owing to the species' life history (although carry‐over parental effects may equally be likely to occur for the reasons previously mentioned). Furthermore, because male and female *A. polyacanthus* are morphologically identical, and the species is monogamous and provides biparental care, we predicted both paternal and maternal effects may be favored.

## 2 METHODS

### 2.1 Study species

*A*. *polyacanthus* is found in shallow waters on coral reefs in the Indo‐Australian archipelago (Robertson, 1973). They form monogamous pairs and breed primarily during summer (Robertson, 1973; Thresher, 1985). Egg clutches are laid in caves with biparental care occurring during embryogenesis and several weeks post‐hatching (Kavanagh, 2000; Robertson, 1973; Thresher, 1985). Post‐hatching parental care is primarily to protect broods from predation. Since *A*. *polyacanthus* lack a dispersal larval stage and adults are site attached with small home ranges (Miller‐Sims et al., 2008; Robertson, 1973), they are unlikely to migrate to more favorable environments under climate warming. This includes moving to deeper waters, which anyway is unlikely to provide relief (i.e., the thermocline is typically much deeper than their maximum depth range; Frade et al., 2018; Jankowski et al., 2015; Lieske & Myers, 1994; Walther et al., 2013). Short‐term elevated temperature has been shown to strongly affect individual *A*. *polyacanthus* performance (Donelson et al., 2010; Munday et al., 2008; Rummer et al., 2014), but biparental effects appear to partially or fully mitigate the negative impacts of elevated temperature on offspring (Donelson et al., 2016; Donelson & Munday, 2015; Donelson, Munday, McCormick, et al., 2012).

### 2.2 Experimental design

**FIGURE 1:**
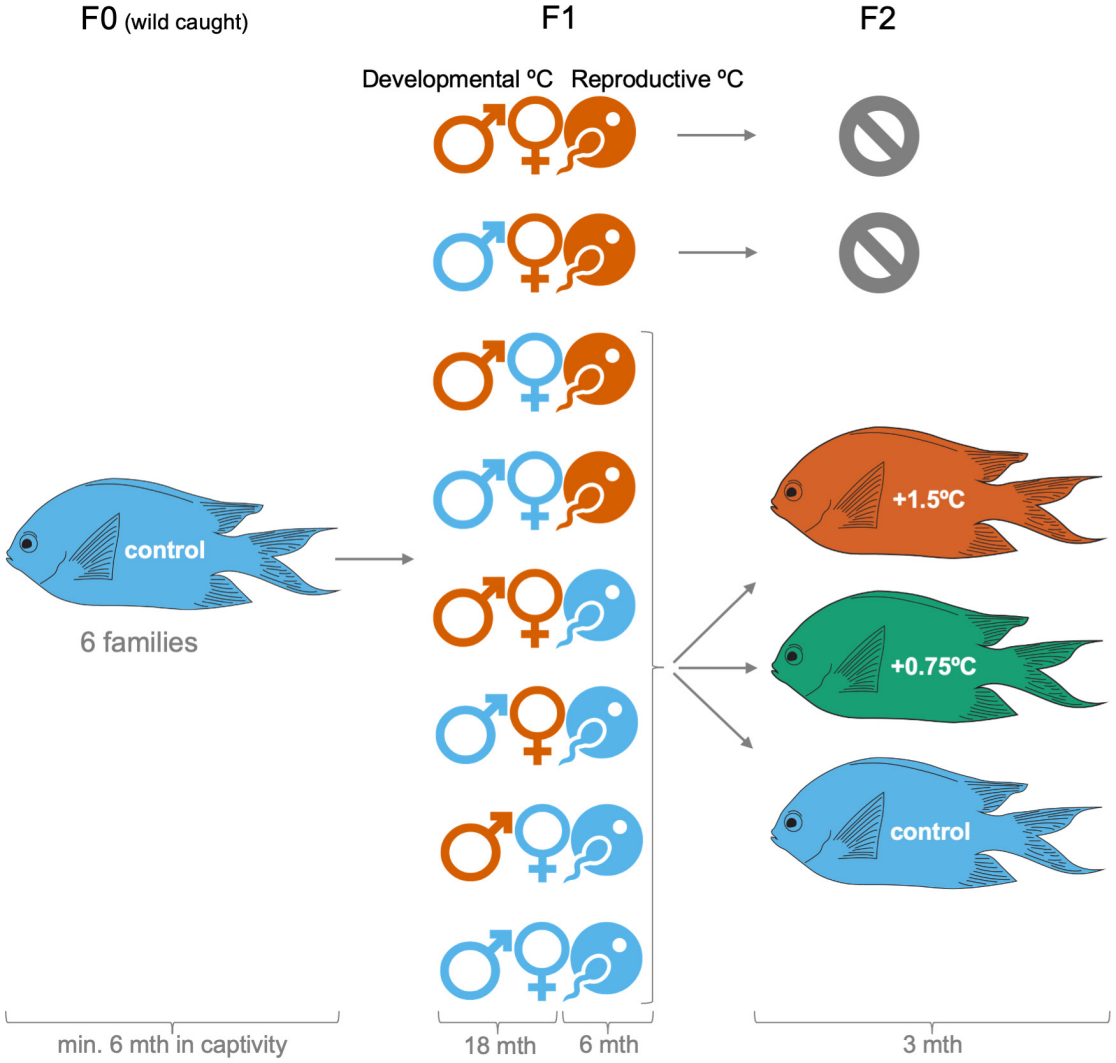
Newly hatched *A. polyacanthus* from six wild‐caught families were split between two developmental temperatures; a present‐day average temperature for their population (control—blue sex symbols) and 1.5°C above the average temperature (orange sex symbols). At maturity, F1 fish were further divided into present‐day control (blue egg and sperm icon) and +1.5°C reproductive temperatures (orange egg and sperm icon). Breeding pairs were created of reciprocal sex crosses of the developmental temperatures across both reproductive temperatures, which resulted in eight F1 parental treatments. 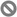 indicates the two F1 treatments that did not reproduce. Newly hatched siblings (F2) were split among a present‐day average summer temperature of 28.5°C (control), 29.25°C (+0.75°C), and 30°C (+1.5°C). Please note that for logistical reasons offspring were kept with their parents until hatching, that is, embryos were exposed to the parent's reproductive temperature

Two generations of *A*. *polyacanthus* were reared in environmentally controlled conditions to examine temperature‐induced parental effects. The experiment began with six wild‐caught pairs from the Palm Islands region (18°37′S, 146°30′E) and nearby Bramble Reef (18°22′S, 146°40′E) of the central Great Barrier Reef to account for genotypic variation (F0 generation, Figure 1). Pairs were kept at seasonally cycling present‐day temperature based on the Palm Islands region and were provided half a terracotta pot as a spawning site. The F0 generation bred in the Austral summer of 2016. Egg clutches were kept with the parents until hatching, allowing them to provide nest care as occurs in the wild. Newly hatched F1 generation siblings were arbitrary divided between a present‐day control and +1.5°C temperature treatment (Figure 1), with 10 fish per tank and a minimum of five replicate tanks per clutch for each temperature treatment. Fish were randomly allocated a tank within their temperature treatment. A 1.5°C increase already occurs on the Great Barrier Reef during marine heatwaves (Frölicher et al., 2018; Hughes et al., 2019; Spinks et al., 2019) and is projected to occur as an average temperature by 2050–2100 (IPCC, 2013). The control water temperature simulated seasonal (winter minimum 23.2°C, summer maximum 28.5°C) and diurnal (03:00 h −0.6°C, 15:00 h +0.6°C) cycles for the Palm Islands region based on temperature loggers from 2002 to 2015 in 0.2–14.6 m depth (AIMS, 2016), with the elevated treatment matching this but 1.5°C higher. Similarly, the photoperiod of the Palm Islands region was replicated with eight fluorescent lamps (36W, 3350L, 3000K, 120 cm tube), reaching a maximum of 13 h 15 min light in summer (December) and a minimum of 11 h 01 min light in winter (June). Seasonal changes to water temperature and illumination were adjusted weekly.

In the Austral winter of 2017, the F1 generation were paired for breeding so that: (1) both males and females developed in control (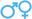), (2) only males developed in +1.5°C (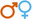), (3) only females developed in +1.5°C (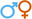), or (4) both males and females developed in +1.5°C (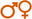). While temperature may impact the timing of maturity, previous work shows *A. polyacanthus* developing in control and +1.5°C temperatures commenced breeding in the same mean month (Donelson et al., 2016). The four pair combinations were further divided into present‐day control (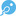) and +1.5°C (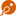) reproductive temperatures, which resulted in eight parental temperature treatments (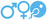, 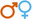, 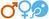, 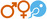, 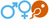, 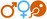, 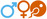, 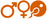; Figure 1). We crossed males and females of one family with another, following Figure 1a in Bonduriansky et al. (2012), such that three family crosses from the original six F0 families were formed. Once breeding pairs were successfully established (see Spinks et al., 2021), the number of replicate pairs per parental treatment inclusive of families was 19 (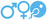), 17 (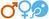), 17 (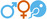), 10 (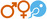), 19 (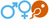), 17 (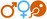), 11 (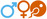), 13 (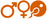). In the Austral summer of 2017/2018, breeding occurred in the F1 generation, although males and females exposed to +1.5°C in both developmental and reproductive life‐stages (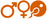) did not breed and only one clutch was produced when males developed in control, females developed in +1.5°C, and reproduction was in +1.5°C (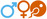; Spinks et al., 2021). This one clutch experienced exceptionally high embryonic mortality (74%) and was excluded from analyses (Spinks et al., 2021). Owing to logistics but also following what occurs in the wild, egg clutches were kept with the parents until hatching. This means for offspring of parents exposed to an elevated reproductive temperature (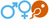 and 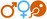), we cannot disentangle the effects of parental reproductive temperature versus early developmental plasticity. Lastly, it is important to note that the hatching data presented in Spinks et al. (2021) were from all first clutches produced during the entire summer breeding season, whereas in this study we present a subset of the clutches that were reared post‐hatching (though results are almost identical). Further details on the F0 and F1 generations and the facilities where they were reared are provided in Spinks et al. (2021).

Newly hatched F2 generation siblings were arbitrary split among a present‐day average summer temperature of 28.5°C (control), 29.25°C (+0.75°C), or 30°C (+1.5°C; Figure 1). Each temperature treatment had a daily temperature cycle of −0.6°C at 03:00 h and +0.6°C at 15:00 hours matching the natural diurnal temperature variation experienced by this population in the wild (AIMS, 2016). For each clutch, siblings were stocked at a density of approximately 20 fish per tank over two replicate tanks for each temperature treatment. This was a higher density with fewer replicate tanks than the F1 generation due to logistical reasons. Hatchlings were randomly allocated a tank within their temperature treatment. A total of 31 clutches were reared to approximately three months of age. We incorporated +0.75°C rearing temperature as it is a halfway point between potentially favorable and unfavorable thermal environments. This F2 rearing treatment that was intermediate to the parental control and +1.5°C temperatures allowed us to observe if (1) any temperature shift b (i.e., an increase or decrease) between generations induced phenotypic change and (2) a smaller temperature increase within and between generations is more beneficial than a larger temperature increase (Donelson et al., 2016). Lastly, by manipulating both parent and offspring environments across a range of ecologically relevant temperatures, we could detect within‐generation plasticity and different types of parental effects (Bonduriansky & Crean, 2018; Donelson et al., 2018).

**FIGURE A1:**
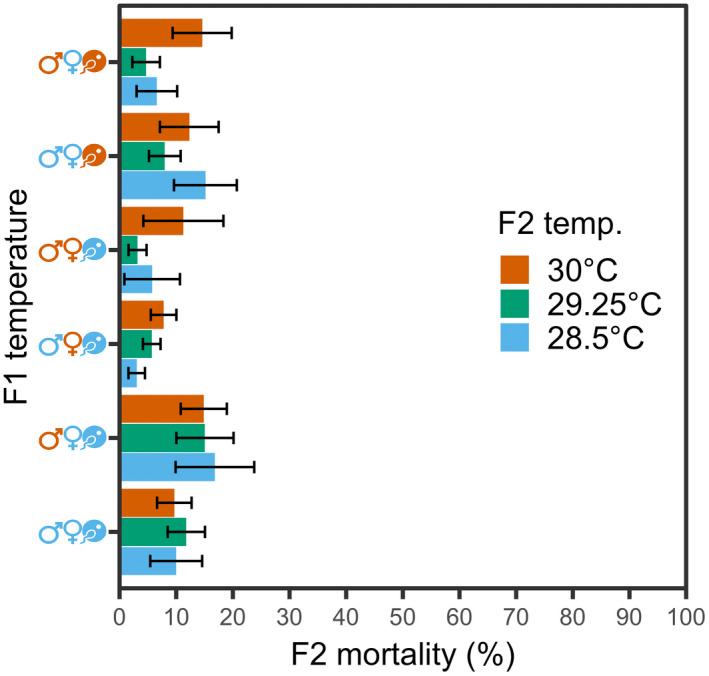
Natural mortality raw mean and standard error of the F2 generation. On the *y* axis, father and mother developmental temperature is represented by sex symbols and the pairs' reproductive temperature by an egg and sperm icon, whereby blue denotes present‐day control temperature and orange a temperature increase of 1.5°C

Hatchlings were given 2–3 h to slowly equilibrate to their rearing temperature via a 2 L tub floated in the tank and receiving gradual inflow. Hatchlings were fed live *Artemia nauplii* the first 6 days (approximately 417 mg dried artemia cysts per tank). On day 4, they began 200–400 μm NRD pellets (INVE Aquaculture) supplied daily at 40 mg tank^−1^. Between days 30 and 59, they were fed 500–800 μm NRD pellets supplied daily at 202 mg tank^−1^ and then on day 60 increased to 404 mg tank^−1^. This is considered a high feeding level (approximately 2% of their body weight at 3 months post‐hatching) for captive *A. polyacanthus* on an energy‐rich formulated diet (Donelson et al., 2010). During rearing, approximately 9% natural mortality occurred (Figure A1). There was also mortality from two incidents of equipment failure (~3% of juveniles); one caused an ammonia spike (~0.25 ppm) and the other oxygen supersaturation, but deaths were evenly spread across treatments and surviving fish did not appear stressed. The F2 generation were maintained in a 15,000 L recirculating system supplied with a continuous flow of natural seawater with precise temperature control (a smaller replica of the facility described in Spinks et al. (2021)). This research was conducted under James Cook University's animal ethics approval A1990, A2210, and A2315.

### 2.3 Measuring size, condition, and sex ratios

Within 12 hours of hatching, approximately 20 offspring (F2 generation) from each clutch to be reared were euthanized by a 1:1 clove oil and ethanol concentrate at 0.125 ml/L of seawater. Hatchlings were then preserved in phosphate‐buffered formaldehyde (4%) and within 48 h weighed (±0.1 mg; excess liquid removed with a Kimwipe) and photographed. Hatch standard length (±0.01 mm) and yolk area (±0.01 mm^2^) were determined from the photographs, by one person (B.L. Spady) who was blinded to the treatments, using ImageJ software v. 1.50i (Schneider et al., 2012). A total of 596 hatchlings were measured. The standard length of 4 hatchlings and the yolk area of 7 hatchlings could not be accurately determined and therefore were excluded.

As described above, from each clutch two replicate tanks of 20 siblings per F2 treatment were grown until approximately three months of age. A total of 3430 juveniles were sexed by external examination of the urogenital papilla in a water‐filled clear bag under a microscope by two experienced researchers (R.K. Spinks and J.M. Donelson; Hilder & Pankhurst, 2003; Robertson, 1973). The juveniles were then euthanized by cervical dislocation, weighed (±1 mg), and their standard length (±0.02 mm) measured. The sex of 42 juveniles, weight of 16 juveniles, and standard length of 12 juveniles could not be accurately determined and, therefore, were excluded. Offspring were sexed and measured specifically between 79 and 106 days post‐hatching (dph; mean 95 dph) due to molecular sampling and swimming performance tests performed in a subset of these fish over this period of time but not presented here. Fulton's K condition factor was calculated as:

$$K=100\frac{W}{L^{3}}$$

whereby *W* is wet weight, *L* is standard length, and the scaling factor is used to bring the condition closer to one (Froese, 2006; Ricker, 1975). Fulton's K condition factor is a widely used proxy for body condition in fishes; nevertheless, it has been criticized (Froese, 2006; Jones et al., 1999; Nash et al., 2006). A common alternative is to model weight as a function of length. During preliminary analysis, we found the weight–length model suffered from outliers, multicollinearity, and Bayesian validation concerns and that the results were identical to Fulton's K condition factor; therefore, we progressed with Fulton's K.

### 2.4 Statistical analysis

Bayesian linear and generalized linear mixed models (LMM and GLMM) were applied using the rstanarm package v. 2.21.1 (Goodrich et al., 2020) in R v. 4.0.3 (R Core Team, 2020). The standard length, weight, or yolk area of newly hatched offspring were dependent variables and modeled with normal distributions (see R script Spinks, 2022). LMMs were validated per Spinks et al. (2019) and followed linear model assumptions of linearity, homogeneity of variances, and normality. Each LMM included F1 temperature (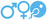, 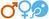, 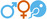, 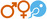, 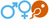, 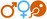) as an independent fixed variable. Each LMM had a random intercept that varied by father family (6 levels) and mother family (6 levels) due to non‐independence between offspring from the same F0 family line of the father and between offspring from the same F0 family line of the mother. The random intercept also varied by F1 pair (30 levels) due to non‐independence between offspring from the same parental pair. Random slopes in addition to random intercepts did not improve the LMM fits visually or based on Bayesian leave‐one‐out cross‐validation (LOO Vehtari et al., 2017).

Offspring standard length, weight, or Fulton's K condition factor at approximately three months of age were dependent variables and modeled with gamma distributions and log links (see R script Spinks, 2022). GLMMs with gamma distributions were better fits as per Spinks et al. (2019) model validation than LMMs with normal distributions where models had to be heavily adjusted and still assumptions of normality were not met and heteroscedasticity was evident in the weight model. However, *r* ^2^ cannot be accurately estimated in a GLMM with a gamma distribution. Each GLMM included the independent fixed variables F1 temperature (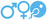, 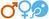, 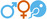, 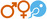, 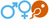, 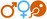), F2 temperature (28.5°C, 29.25°C, 30°C), their interactions, and the covariates of offspring age and density (centered and scaled). These covariates were included because offspring were measured between 79 and 106 dph (mean 95) and the tank density varied between 4 and 31 fish (mean 20) owing to small clutches, deaths, or miscounting. Significant interactions or collinearity were not present between these covariates and the range of offspring ages and densities overlapped in the F1 and F2 temperature treatments. Finally, sex ratio was a dependent variable and modeled with a binomial distribution and log odds link (see R script Spinks, 2022). The independent fixed variables were F1 temperature (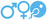, 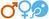, 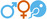, 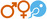, 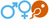, 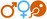), F2 temperature (28.5°C, 29.25°C, 30°C), and their interactions.

Offspring standard length, weight, Fulton's K condition factor, and sex ratio models at three months had the same random effects structure owing to the hierarchical nature of the experimental design and to avoid pseudoreplication. Each model's random intercept varied by father family (6 levels) and mother family (6 levels) due to non‐independence between offspring from the same F0 family line of the father and between offspring from the same F0 family line of the mother. The random intercept also varied by F2 rearing tank (181 levels) nested in F1 pair (30 levels) due to non‐independence between offspring from the same tank and offspring from the same parental pair. Random slopes in addition to random intercepts did not improve the model fits visually or based on LOO. Variation attributed to random effects is stated in the link scale.

Bayesian models allow the integration of prior knowledge (van de Schoot et al., 2021). We specified weakly informative priors using rstanarm (Table A1; see R script Spinks, 2022). The posterior distribution is derived from the priors (previous evidence) and the likelihood function (new evidence; van de Schoot et al., 2021). Visual posterior checks confirmed that priors never heavily influenced the posterior. Using the Hamiltonian Monte Carlo algorithm, models were run with three chains by means of the No‐U‐Turn sampler for a minimum 5000 iterations with at least every second posterior sample thinned and a minimum of 40% discarded depending upon the complexity of the model (see R script Spinks, 2022). Bayesian model validation followed Spinks et al. (2019). In order to compare among parental temperatures without confounding offspring rearing temperature effects, groups were compared with their respective offspring rearing temperature (28.5°C, 29.25°C, or 30°C) of control parents (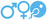). Statistical significance was determined by the probability of the posterior density distribution being greater or lesser than the comparison (i.e., zero; see R script Spinks, 2022). Posterior probabilities are expressed as a percent with a higher or lower value suggesting greater confidence in a group being different from its comparison, whereas nearer to 50% suggests little confidence in a group being different from its comparison. Note that Bayesian inference (with suitable priors) does not require correction for multiple comparisons (Gelman & Tuerlinckx, 2000). Figures were created with the R packages' emmeans v. 1.5.1 (Lenth, 2020) and tidybayes v. 2.1.1 (Kay, 2020). The data and R code needed to replicate these analyses are available in Spinks (2022).

## 3 RESULTS

### 3.1 Maternal exposure to warming produced hatchlings with larger yolks whilst reproductive exposure decreased hatchling length and weight

**FIGURE 2:**
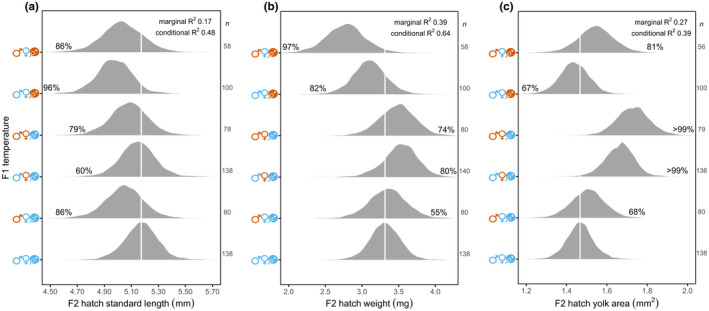
Entire Bayesian posterior density distributions of offspring (a) standard length, (b) weight, and (c) yolk area at hatching from each parental temperature treatment. On the *y* axes, father and mother developmental temperature is represented by sex symbols and the pairs' reproductive temperature by an egg and sperm icon, whereby blue denotes present‐day control temperature and orange a temperature increase of 1.5°C. Posterior probabilities (i.e., confidence in an effect) are shown to the left of the vertical white line (intercept) when smaller in size or to the right of the line when larger in size relative to hatchlings of control parents (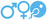). Sample size (*n*) is number of hatchlings

Parental reproductive temperature had a greater overall effect on newly hatched offspring length and weight than did parental developmental temperature (Figure 2a,b). By contrast, maternal developmental temperature affected newly hatched offspring yolk reserves (Figure 2c). Hatchlings of control parents (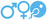) had a median 5.17 mm standard length and 3.31 mg weight with 1.47 mm^2^ yolk area. Hatchlings of parents where the father, mother, or both parents developed in +1.5°C, but reproduction occured in control temperatures (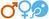, 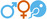, or 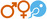), were similar in length and weight compared with hatchlings of control parents (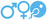; Figure 2a,b). When mothers developed in +1.5°C (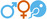 and 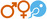), hatchlings had a median 14% and 18% larger yolk area than progeny from control parents (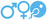; Figure 2c). When both parents developed in control temperatures, but reproduced in +1.5°C (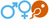), their hatchlings were a median 4% shorter compared with hatchlings of control parents (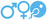; Figure 2a), but they were similar in weight (Figure 2b). When fathers developed in +1.5°C, mothers developed in control temperature, and reproduction was in +1.5°C (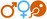), hatchlings were more similar in length to hatchlings of control parents (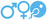; Figure 2a) and to pairs where the father developed in +1.5°C, but reproduced in control temperature (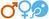). However, these hatchlings (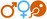) were a median 16% lighter compared with hatchlings of control parents (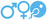; Figure 2b) and a median 17% lighter compared with hatchlings of fathers that developed in +1.5°C and reproduced in control conditions (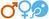; 96% probability of weighing less).

Variation attributed to paternal and maternal family‐level effects was less than the magnitude of parental temperature effects (standard length σ 0.02 mm, weight σ 0.03 mg, and yolk area σ 0.01 mm^2^). Variation attributed to parental pair was equivalent to or lower than family‐level effects for standard length and yolk area but slightly higher for weight (σ 0.1 mg).

### 3.2 Offspring reared in warmer water were shorter and in higher condition

**FIGURE 3:**
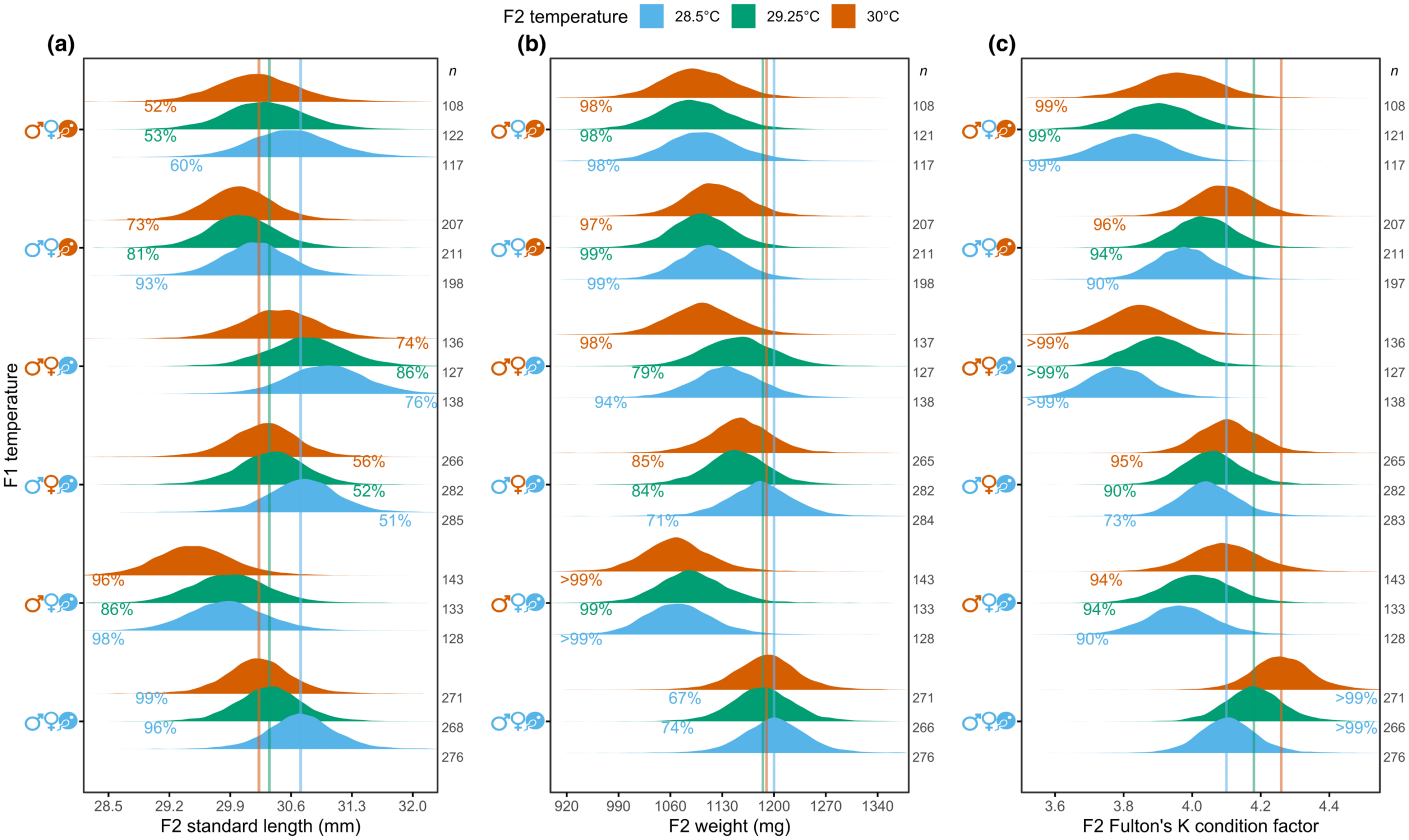
Entire Bayesian posterior density distributions of offspring (a) standard length, (b) weight, and (c) Fulton's K condition factor at the average age of 95 days post‐hatching and density of 20 fish per tank for each parental and offspring temperature treatment. On the *y* axes, father and mother developmental temperature is represented by sex symbols and the pairs' reproductive temperature by an egg and sperm icon, whereby blue denotes present‐day control temperature and orange a temperature increase of 1.5°C. Posterior probabilities (i.e., confidence in an effect) are colored blue when compared to offspring reared in 28.5°C of control parents (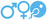; vertical blue lines), green when compared to offspring reared in 29.25°C of control parents (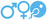; vertical green lines), or orange when compared to offspring reared in 30°C of control parents (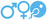; vertical orange lines). Probabilities to the left of the vertical lines indicate smaller size/condition relative to the comparison, whereas probabilities to the right of the vertical lines indicate larger size/condition relative to the comparison. Sample size (*n*) is number of offspring

When parents developed and reproduced in control temperature (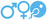) and their offspring were reared for three months in warmer water, juveniles were shorter, but had the same weight and thus were in higher condition (Figure 3). At the average age (95 dph) and density (20 fish), offspring of control parents (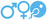) reared in 28.5°C had a median 30.70 mm standard length, 1200 mg weight, and 4.10 Fulton's K condition factor. When sibling offspring were instead reared in 29.25°C or 30°C, they were a median 1% or 2% shorter, respectively, compared with offspring reared in 28.5°C (Figure 3a). Since weight did not differ for offspring of control parents (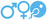) among rearing temperatures (28.5°C, 29.25°C or 30°C; Figure 3b), offspring reared in 29.25°C or 30°C were in higher condition by a median of 2% or 4%, respectively, relative to offspring reared in 28.5°C (Figure 3c).

### 3.3 Parental exposure to warming decreased offspring weight and condition

The father's developmental temperature affected offspring length, weight, and condition. Offspring reared in 28.5°C from fathers developed in +1.5°C, mothers developed in control, and reproduction in control temperature (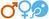) were a median 3% shorter, 11% lighter, and 3% lower in condition compared with offspring reared in 28.5°C of control parents (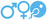; Figure 3a–c). Sibling offspring reared in 29.25°C were a median 2% shorter, although there was less certainty in this trend, 8% lighter, and 4% lower in condition compared with offspring reared in 29.25°C from control parents (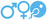; Figure 3a–c). When reared in 30°C, sibling offspring were a median 2% shorter, 10% lighter, and 4% lower in condition compared with offspring reared in 30°C of control parents (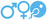; Figure 3a–c).

The mother's developmental temperature affected offspring weight and condition, but the effects were less marked than for the father's developmental temperature alone (above). Offspring from fathers developed in control, mothers developed in +1.5°C, and reproduction in control temperature (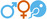) were similar in length across their rearing temperatures compared with offspring of control parents (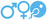) in those same rearing temperatures (Figure 3a). Offspring reared in 28.5°C were similar in weight and condition relative to offspring reared in 28.5°C of control parents (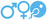; Figure 3b,c). However, there was a trend of sibling offspring reared in 29.25°C or 30°C weighing a median 3% or 7% less, respectively, compared with offspring of control parents (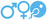) in those same offspring developmental temperatures (Figure 3b). Further, an interaction was present between mother and offspring temperatures, with offspring reared in 29.25°C or 30°C a median 3% or 4% lower in condition, respectively, compared with offspring of control parents (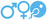) in those same rearing temperatures (Figure 3c).

Both parent's developmental temperature affected offspring weight and condition. Offspring from fathers and mothers that developed in +1.5°C, but reproduced in control temperature (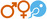), were similar in length when reared in 28.5°C and 30°C compared with offspring of control parents (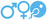) in those same rearing temperatures (Figure 3a). However, offspring reared in 28.5°C were a median 11% lighter and 8% lower in condition compared with offspring reared in 28.5°C of control parents (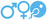; Figure 3b,c). Sibling offspring reared in 29.25°C tended to be lighter by a median 3%, which resulted in a median 7% lower condition compared with offspring reared in 29.25°C of control parents (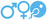; Figure 3a–c). Sibling offspring reared in 30°C were a median 7% lighter and 10% lower in condition compared with offspring reared in 30°C of control parents (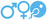; Figure 3b,c).

The parent's reproductive temperature affected offspring length, weight, and condition. Offspring reared in 28.5°C from fathers and mothers that developed in control temperature but with reproduction in +1.5°C (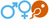) were a median 2% shorter, 7% lighter, and 3% lower in condition compared with offspring reared in 28.5°C of control parents (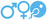; Figure 3a–c). When sibling offspring were instead reared in 29.25°C, their standard length was similar, but they were a median 7% lighter and 4% lower in condition compared with offspring reared in 29.25°C of control parents (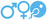; Figure 3a–c). Sibling offspring reared in 30°C were of similar length, but were a median 6% lighter and 4% lower in condition compared with offspring reared in 30°C of control parents (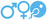; Figure 3a–c).

The father's developmental and reproductive temperature affected offspring weight and condition. Offspring from fathers developed in +1.5°C, mothers developed in control, and with reproduction in +1.5°C (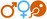) were similar in length, irrespective of their rearing temperature, compared with offspring of control parents (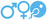) in those same rearing temperatures (Figure 3a). Offspring reared in all temperatures (28.5°C, 29.25°C, and 30°C) were a median 8% lighter and 7% lower in condition compared with offspring of control parents (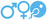) in those respective rearing temperatures (Figure 3b,c).

Comparing offspring from pairs with fathers developing in +1.5°C and mothers developing in control, and with reproduction either in control (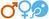) or +1.5°C temperatures (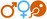), showed little difference in offspring weight (probabilities ≤74%). However, offspring reared in 28.5°C and 30°C from fathers continuously exposed to +1.5°C (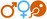) were a median 2% longer compared with offspring where fathers were only exposed to +1.5°C in development (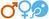; both 93% probability of longer length). Accordingly, this resulted in trends of lower condition by a median 3% for offspring reared at 28.5°C and 30°C compared with offspring of parents reproducing in control (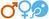) reared at those temperatures (87% and 85% probability of lower condition). Offspring from fathers continuously exposed to +1.5°C (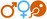) reared in 29.25°C were similar in length and condition to offspring reared in 29.25°C of parents reproducing in control temperature (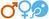; probabilities ≤83%).

Comparing offspring from pairs with both parents developing in control temperature and with reproduction in +1.5°C (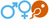) or with fathers developing in +1.5°C (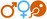) showed little difference in offspring length or weight (probabilities ≤81%). However, offspring reared in all temperatures (28.5°C, 29.25°C, and 30°C) from fathers continuously exposed to +1.5°C (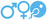) showed trends of lower condition by a median 3–4% compared with offspring from fathers developing in control (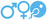) in the respective rearing temperatures (86%–89% probability of lower condition).

**FIGURE A2:**
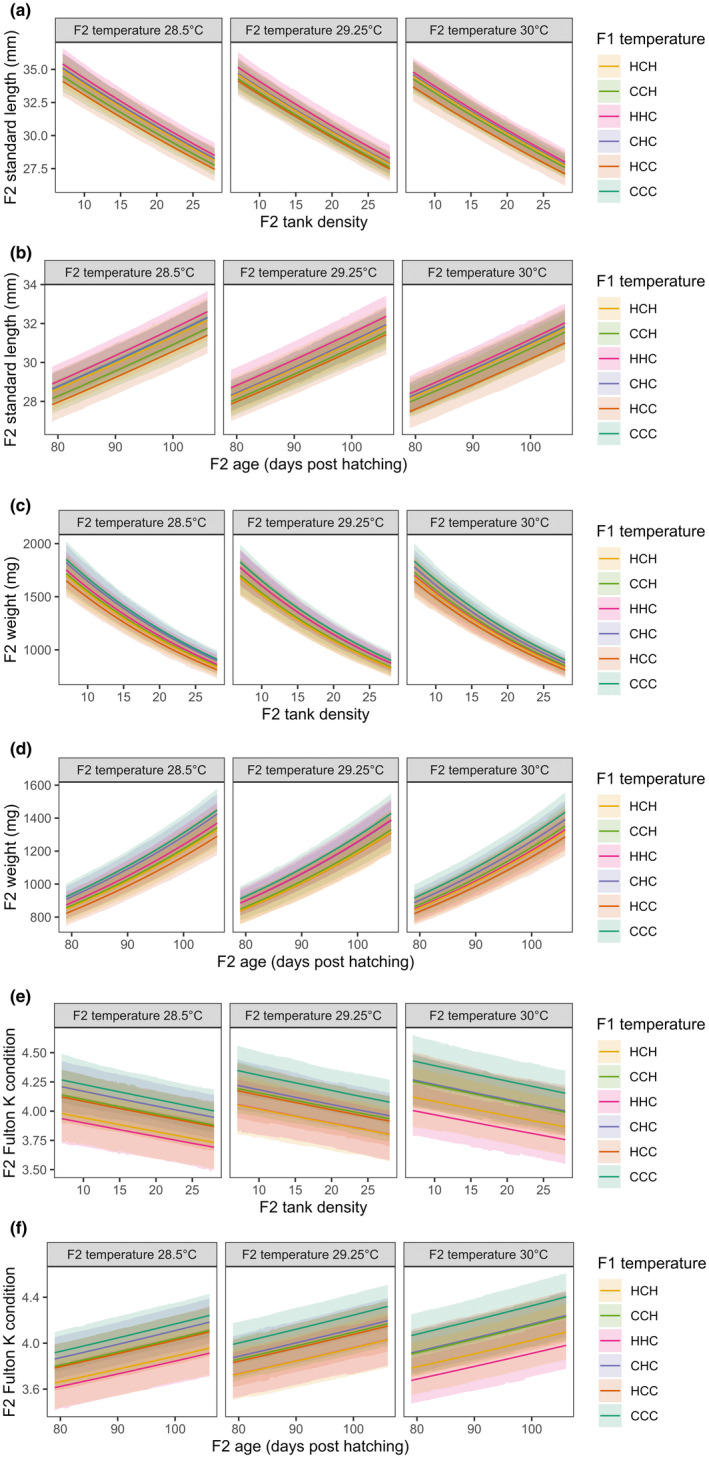
Modeled relationship of size/condition and the number of fish per tank or age in the F2 generation. Bayesian posterior medians (solid lines) with 95% credible intervals (ribbons) of fish (a) standard length and density, (b) standard length and age, (c) weight and density, (d) weight and age, (e) Fulton's K condition factor and density, and (f) Fulton's K condition factor and age, for each F1 and F2 temperature treatment. F1 treatment codes are defined by C representing control temperature and H representing +1.5°C with the first letter indicating the father's developmental temperature, the second letter the mother's developmental temperature, and the third letter the pair's reproductive temperature

**FIGURE A3:**
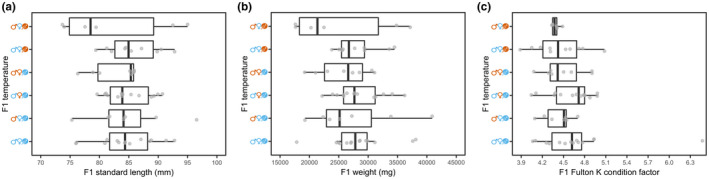
Size and condition raw values of the F1 generation. Medians (thick lines), first and third quartiles (hinges), and values no more than 1.5 times the inter‐quartile range (whiskers) of parental (a) standard length, (b) weight, and (c) Fulton's K condition factor for each temperature experience. On the *y* axis, father and mother developmental temperature is represented by sex symbols and the pairs' reproductive temperature by an egg and sperm icon, whereby blue denotes present‐day control temperature and orange a temperature increase of 1.5°C. Grey points are individual fish. Only parents whose offspring were reared (i.e., presented in this paper) are shown. Parents were measured approximately after two clutches had been laid in the 2017/2018 summer breeding season (~2 years of age), although for logistical reasons not all parents could be measured

Offspring standard length, weight, and Fulton's K condition factor decreased as fish density increased and increased as fish aged (Figure A2). Variation attributed to paternal and maternal family‐level effects was less than the magnitude of parental and offspring temperature effects for standard length (σ 0.0002 log vs largest treatment effect −0.03 log), weight (σ 0.002 log vs largest treatment effect −0.1 log), and Fulton's K condition factor (σ 0.0003 log vs largest treatment effect −0.08 log). Variation attributed to parental pair and offspring rearing tank was equivalent to or lower than family‐level effects. Lastly, the parental generation's standard length, weight, and Fulton's K condition factor showed no clear pattern among treatments (Figure A3).

### 3.4 Offspring rearing or parental temperature had little influence on offspring sex ratios

Offspring reared for three months in 28.5°C from control parents (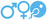) had a median 0.53 proportion male, as expected (Table 1). Offspring sex ratios were skewed in some treatments, for example, the most consistent and largest effects were decreases in males in all offspring rearing temperatures when fathers developed in +1.5°C, mothers developed in control, and reproduction was in control temperature (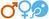) compared with the respective offspring rearing temperatures of control parents (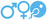; Table 1). However, parent and offspring temperatures only explained 2% of the total variation in sex ratios (i.e., marginal *r* ^2^). Furthermore, only 4% of the total variation in sex ratios was explained when also including the random effects (i.e., conditional *r* ^2^) such as paternal and maternal family effects, parental pair, and offspring rearing tank.

**TABLE 1:** Offspring sex ratios

F1 temperature	F2 temperature (°C)	*n*	Median proportion male	95% CI proportion male	Probability male bias (%)	Probability female bias (%)
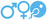	28.5	263	0.53	0.42–0.62	NA	NA
	29.25	264	0.49	0.39–0.59	18	82
	30	271	0.58	0.49–0.68	88	12
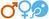	28.5	128	0.45	0.32–0.57	13	87
	29.25	133	0.42	0.30–0.54	18	82
	30	142	0.41	0.29–0.54	1	99
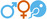	28.5	287	0.49	0.39–0.60	29	71
	29.25	279	0.50	0.38–0.60	56	44
	30	264	0.57	0.47–0.67	43	57
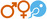	28.5	140	0.51	0.39–0.63	39	61
	29.25	127	0.56	0.42–0.69	84	16
	30	137	0.53	0.41–0.66	23	77
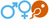	28.5	197	0.51	0.40–0.62	38	62
	29.25	210	0.44	0.33–0.55	25	75
	30	200	0.55	0.44–0.66	28	72
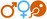	28.5	116	0.64	0.52–0.78	92	8
	29.25	121	0.53	0.40–0.66	73	27
	30	109	0.55	0.41–0.68	35	65

*Note*: The fourth and fifith columns show Bayesian posterior medians and 95% highest posterior density credible intervals (CI) are shownof offspring proportion male at approximately three‐months post‐hatching for each parental (F1) and offspring (F2) temperature. The sixth and seventh columns show posterior probabilities (i.e., confidence) of a male or female bias, expressed as a percent, with the comparison to the respective offspring rearing temperature (28.5°C, 29.25°C, or 30°C) of control parents (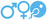). Within control parents (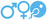), the posterior probabilities for offspring reared in 29.25°C and 30°C are relative to sibling offspring reared in 28.5°C. Father and mother developmental temperature is represented by sex symbols and the parental reproductive temperature by an egg and sperm icon whereby blue denotes present‐day control temperature and orange a temperature increase of 1.5°C. Sample size (*n*) is number of offspring.

## 4 DISCUSSION

Our results show that juvenile growth in a coral reef fish is affected by elevated rearing temperatures and elevated developmental and reproductive temperatures experienced by the mother and father. For offspring whose parents were exposed to present‐day control temperature, a warmer rearing temperature reduced standard length compared with siblings reared in control temperature. However, these warm‐reared juveniles maintained their weight, which resulted in a higher body condition. Higher physical condition may increase predator evasion, competitive ability, and thermal tolerance and, therefore, could be adaptive (Booth & Hixon, 1999; Grorud‐Colvert & Sponaugle, 2006; Poulos & McCormick, 2015; Robinson et al., 2008). By contrast, parental exposure to warming, irrespective of ontogenetic timing and sex, resulted in lighter and lower condition offspring in all rearing temperatures, that is, carry‐over parental effects. Reduced weight and condition are generally thought to be maladaptive (Booth & Beretta, 2004; Booth & Hixon, 1999; Goatley & Bellwood, 2016; Grorud‐Colvert & Sponaugle, 2006; Meekan et al., 2006), however, combined with previous studies in *A*. *polyacanthus* they could be the result of an adaptive parental effect on metabolism (Donelson, Munday, McCormick, et al., 2012; Munday et al., 2016). Consequently, warm‐exposed parents may produce offspring that maintain their metabolic rate in ambient elevated temperatures, but at a cost of reduced weight and subsequent loss of condition since length was typically maintained. Conversely, offspring sex ratios were not strongly influenced by their rearing temperature or that of their parents. Importantly, family‐level effects were minimal in all traits, indicating that the observed phenotypic changes in the present study are unlikely to be the result of differential performance among genotypes. These results show the overriding influence of parental effects and highlight the potential trade‐offs of plasticity within and between generations.

Within‐generation plasticity resulted in slightly shorter fish that maintained their weight and accordingly were in better condition with increasing temperature. Metabolic rates of ectotherms increase with rising temperature (Gillooly et al., 2001; Pörtner & Knust, 2007). Given that the energetic resources (e.g., yolk provisioning and food) were equal across offspring rearing temperatures from control parents, it seems that standard length was sacrificed while weight was maintained, thus increasing physical condition. Our results suggest plasticity scales with temperature, as the length and condition of offspring reared in +0.75°C (29.25°C) were halfway between those phenotypes in sibling offspring reared in control (28.5°C) and +1.5°C (30°C) temperatures. Increasing physical condition with warming during development has been observed previously in *A*. *polyacanthus* and other damselfishes (Donelson, 2015; Donelson et al., 2014; Donelson, Munday, McCormick, et al., 2012; Grenchik et al., 2013). Furthermore, natural latitudinal thermal gradients show that as water temperature increases above ~28.5°C, larval growth and length at settlement decrease in some reef fishes (McLeod et al., 2014). However, maintenance of condition may not be a consistent pattern across reef fishes, as wrasses reared in warmer water had reduced length, weight, and body condition (Motson & Donelson, 2017). It may be that for some reef fishes, high condition is beneficial in elevated temperatures as it can increase predator evasion, competitive ability, and enhance thermal tolerance (Booth & Beretta, 2004; Booth & Hixon, 1999; Grorud‐Colvert & Sponaugle, 2006; Robinson et al., 2008); thus, this within‐generation plasticity could be adaptive. Since food availability can influence the impact of temperature (Donelson et al., 2010; Donelson, Munday, & McCormick, 2012; Munday et al., 2008), it is likely that by providing juveniles in this experiment with ample food it allowed the observed maintenance of weight and increasing condition. Maintenance of weight and physical condition may be more variable in natural populations compared with the laboratory experiments conducted here due to the temporal and spatial variation in food supply in the wild, especially as the oceans warm (Munday et al., 2009).

Parental effects were observed with parental exposure to warming decreasing offspring weight and condition relative to offspring of parents exposed solely to present‐day temperature. Reduced offspring weight and physical condition at three months post‐hatching were observed regardless of when the parents were exposed to warming (development and/or reproduction) and whether the mother, father, or both parents were exposed. The parental effects were also similar across offspring rearing temperatures, which suggests they are carry‐over effects (Bonduriansky & Crean, 2018; Jablonka et al., 1995). Since carry‐over parental effects are assumed to be widespread across taxa (Bonduriansky & Crean, 2018; Jablonka et al., 1995), we are unsurprised by this outcome; however, there was also an expectation of anticipatory parental effects. This is because *A. polyacanthus* parental environment should be predictive of the offspring environment, as offspring stay with their parents for several weeks post‐hatching and adults rarely disperse from their natal reef (Miller‐Sims et al., 2008). Furthermore, there is evidence of anticipatory parental effects in *A. polyacanthus* in other traits (Donelson & Munday, 2015). Quantifying temperature predictability across generations in this species will be an important next step to confirm our expectations (Burgess & Marshall, 2014).

A lower body weight may be considered adaptive in water‐breathing animals due to an increase in heat tolerance (Forster et al., 2012; Leiva et al., 2019), but this is dependent on maintaining body condition (Robinson et al., 2008). This was not the case for progeny from warm‐exposed parents. Therefore, it seems unlikely that lighter and lower condition juveniles had higher heat tolerance. Furthermore, lighter and lower condition individuals may have a higher predation risk and reduced competitive ability (Booth & Beretta, 2004; Booth & Hixon, 1999; Goatley & Bellwood, 2016; Grorud‐Colvert & Sponaugle, 2006; Meekan et al., 2006; Poulos & McCormick, 2015; Shima & Swearer, 2010). Alternately, the decrease in offspring weight and subsequent decline in condition may be genetically linked to an adaptive parental effect on metabolism. Previous studies have shown that *A. polyacanthus* offspring from warm‐exposed parents increased their maximum metabolic rate and thus restored their aerobic scope at elevated temperatures and both these traits showed negative genetic correlations with body weight (Donelson, Munday, McCormick, et al., 2012; Munday et al., 2016). Together, these results illustrate the complex trade‐offs between traits that can occur and the difficulty of identifying the potential adaptive or maladaptive nature of plastic changes.

Differences in offspring weight at hatching did not simply carry through to three months of age, as hatching weight was similar in all parental groups that reproduced at control temperature. When parents reproduced at +1.5°C, newly hatched offspring were either shorter or lighter, which is not surprising given embryos developed in the same elevated temperature as their parents and warming can increase metabolic and developmental rates (Sheridan & Bickford, 2011; Spinks et al., 2021). Alternatively, smaller hatchlings may be the result of stressed parents devoting less energy to embryonic care (Spatafora et al., 2021; Wiley & Ridley, 2016). Nevertheless, by three months post‐hatching it did not seem to matter whether parents had been exposed to higher temperature during development or reproduction; offspring were lower in weight and condition. Development has been previously revealed as a crucial period to induce beneficial plasticity within and between generations (Donelan et al., 2020; Donelson & Munday, 2015; Spinks et al., 2019, 2021; West‐Eberhard, 2003), yet our findings suggest the ontogenetic timing of exposure to warming in the parental generation does not have a substantive effect (positive or negative) on offspring weight and condition at three months post‐hatching. However, fathers with combined developmental and reproductive exposure to warming produced slightly poorer condition offspring compared with offspring of fathers with either developmental or reproductive exposure to warming. An additive effect of combined developmental and reproductive exposure to warming was also observed in terms of a substantial decline in reproduction from females and reduced reproductive output from males (Spinks et al., 2021), suggesting continued warming could significantly impact population sustainability.

While any exposure to warming of parents generally reduced offspring growth, there were some differences in the magnitude depending on the paternal and maternal timing of exposure. Interestingly, pairs where fathers developed in +1.5°C, mothers developed in present‐day temperature, and reproduction was in present‐day temperature, offspring were shorter at three‐month post‐hatching, in addition to decreased weight and condition. This paternal effect is likely a trade‐off, similar to that observed for within‐generation plasticity, whereby individual length is reduced to lessen the impact on physical condition. Evidence for environment‐induced paternal effects on offspring size is increasing (e.g., Bonduriansky & Head, 2007; Northstone et al., 2014; Shama & Wegner, 2014). For instance, low‐condition male guppies were shown to produce poor‐quality sperm and consequently had smaller‐sized offspring (Evans et al., 2017). Since *A. polyacanthus* exhibits biparental care, there may be even greater opportunity for fathers to influence offspring development and condition. A reduction in anemone fish paternal fanning, for example, decreased ambient dissolved oxygen in the egg clutch, and this could impact embryonic metabolism and developmental success (Green & McCormick, 2005b). However, we did not observe the same reduction in juvenile length when +1.5°C fathers paired with control mothers and instead reproduced in +1.5°C. The only other parental group where there was a reduction in offspring length at three months was mothers and fathers that developed in present‐day control temperature but reproduced in +1.5°C, although this was likely due to offspring hatching at a shorter length.

Exposure of offspring or parents to warming did not strongly skew offspring sex ratios. Offspring reared in present‐day temperature from parents exposed solely to present‐day temperature produced the expected 1:1 sex ratio for *A*. *polyacanthus*. Intriguingly, we observed no sex bias when siblings were reared at 0.75°C and 1.5°C above summer average temperatures from hatching. Mixed results of the impact of developmental warming on sex determination have been observed in populations of *A*. *polyacanthus* from similar collection locations. Specifically, a significant male bias was found when fish were reared from hatching in +1.5°C (mean proportion males 0.66) and +3°C (mean proportion males 0.72 and 0.90) (Donelson & Munday, 2015; Rodgers et al., 2017); however, in other experiments no sex bias was observed in +1.5 and +2°C rearing treatments (Rodgers et al., 2017; Spinks et al., 2019). Given previous and current findings, it seems likely that a thermal threshold of sex bias exists around 1.5°C above present‐day temperature, which may vary genetically within and among populations of *A. polyacanthus*. This is not surprising since sex can be determined by an interaction of genetics and environmental temperature in fishes (Ospina‐Álvarez & Piferrer, 2008). Similarly, parents exposed to warming generally had little influence on offspring sex ratios. The only consistent trend was slightly more daughters by fathers that developed in +1.5°C, mothers that developed in present‐day control temperature, and reproduction in control temperature. In anole lizards, poorer condition fathers have produced female‐biased offspring (Cox et al., 2011). Sex allocation theory predicts that parents in poor condition should invest in the sex that is less costly to produce or the sex that results in enhanced fitness in those conditions (Trivers & Willard, 1973). Yet, *A*. *polyacanthus* are sexually monomorphic, so it is difficult to think of a sex‐specific cost or advantage and while offspring were smallest from this parental treatment group, there was not clear indication that the fathers were in poorer condition (Figure A3). Furthermore, parental and offspring temperatures combined only explained a small amount (2%) of the total variation in offspring sex ratios, suggesting that any bias was stochastic and not actually driven by the temperature treatments (or family, pair, and offspring rearing tank as these only explained a further 2% of total variation).

Global warming appears to be shrinking ectotherms (Geffroy & Wedekind, 2020; Reading, 2007; Sheridan & Bickford, 2011). Our findings in a coral reef fish support this and further show that shrinking is exacerbated by parental effects. Smaller size may be the result of a trade‐off with metabolism between generations (Donelson, Munday, McCormick, & Pitcher, 2012; Munday et al., 2016; Pettersen et al., 2019) but could allow *A*. *polyacanthus* to adjust to elevated sea temperatures rapidly. The potential fitness benefits of this trade‐off certainly warrant further investigation in this species. We are also the first to demonstrate subtle differences in offspring growth in a tropical ectotherm due to the timing of maternal, paternal and biparental exposure to warming. These findings highlight the multiple potential mechanisms behind parental effects as well as emphasize the need for more studies to consider the fathers’ influence on offspring size. By contrast, sex ratios were typically not influenced by offspring or parental elevated temperatures, and these findings, combined with previous work, may suggest the threshold of sex bias in this species is around 1.5°C above summer average temperature and interacts with genetic sex determination. Together, our findings show that in a warm ocean, within‐generation plasticity and parental effects can influence individual performance and result in trade‐offs between traits, all of which may translate to effects on population sustainability.

## AUTHOR CONTRIBUTIONS

**Jennifer M. Donelson:** Conceptualization (lead); funding acquisition (supporting); investigation (supporting); methodology (supporting); project administration (equal); resources (supporting); supervision (equal); validation (equal); writing – original draft (supporting); writing – review and editing (equal). **Lucrezia C. Bonzi:** Methodology (supporting); project administration (supporting); writing – review and editing (supporting). **Philip L. Munday:** Conceptualization (equal); formal analysis (supporting); funding acquisition (equal); investigation (supporting); project administration (supporting); resources (lead); supervision (equal); validation (equal); writing – original draft (supporting); writing – review and editing (equal). **Rachel K. Spinks:** Conceptualization (equal); data curation (lead); formal analysis (lead); funding acquisition (supporting); investigation (lead); methodology (lead); project administration (equal); resources (supporting); validation (lead); visualization (lead); writing – original draft (lead); writing – review and editing (lead). **Timothy Ravasi:** Conceptualization (supporting); funding acquisition (lead); resources (supporting); writing – review and editing (supporting).

## CONFLICT OF INTEREST

The authors declare there are no conflicts of interest.

### OPEN RESEARCH BADGES

This article has earned an Open Materials badge for making publicly available the components of the research methodology needed to reproduce the reported procedure and analysis. All materials are available at https://doi.org/10.25903/v4kc‐3a19.

## Data Availability

The R code used to generate the analyses presented in the paper and the associated data are available from Research Data James Cook University DOI: 10.25903/v4kc‐3a19, https://doi.org/10.25903/v4kc‐3a19 (Spinks, 2022).

## APPENDIX A

**TABLE A1:** Default weakly informative priors of rstanarm v. 2.21.1 used in the models

Model	Intercept	Slope	Error standard deviation
Hatch length	*Normal* (5.2, 0.77 mm)	*Normal* (0, [2.25, 1.82, 2.27, 2.05, 2.59] mm)	*Exponential* (rate 3.2)
Hatch weight	*Normal* (3.4, 1.4 mg)	*Normal* (0, [4.16, 3.34, 4.16, 3.79, 4.78] mg)	*Exponential* (rate 1.8)
Hatch yolk area	*Normal* (1.6, 0.61 mm^2^)	*Normal* (0, [1.77, 1.44, 1.78, 1.61, 2.06] mm^2^)	*Exponential* (rate 4.1)
3mths length	*Normal* (0, 2.5 log)	*Normal* (0, [7.74, 5.82, 7.77, 6.50, 8.27, 5.30, 5.31, 2.50, 2.50, 12.92, 9.08, 13.21, 10.38, 13.47, 12.48, 9.33, 12.78, 10.48, 14.29] log)	*Exponential* (rate 1)
3mths weight	*Normal* (0, 2.5 log)	*Normal* (0, [7.74, 5.82, 7.75, 6.50, 8.28, 5.30, 5.31, 2.50, 2.50, 12.91, 9.08, 13.20, 10.38, 13.52, 12.47, 9.34, 12.73, 10.47, 14.28] log)	*Exponential* (rate 1)
3mths Fulton's K	*Normal* (0, 2.5 log)	*Normal* (0, [7.73, 5.82, 7.76, 6.50, 8.28, 5.30, 5.31, 2.50, 2.50, 12.91, 9.07, 13.20, 10.37, 13.51, 12.47, 9.34, 12.77, 10.47, 14.27] log)	*Exponential* (rate 1)
Sex ratio	*Normal* (0, 10 log odds)	*Normal* (0, 2.5 log odds)	Not applicable

The prior distributions are provided in italics and the prior means and standard deviations in round brackets unless otherwise specified. The prior slope provides a standard deviation for each coefficient in square brackets.
